# ccImpute: an accurate and scalable consensus clustering based algorithm to impute dropout events in the single-cell RNA-seq data

**DOI:** 10.1186/s12859-022-04814-8

**Published:** 2022-07-22

**Authors:** Marcin Malec, Hasan Kurban, Mehmet Dalkilic

**Affiliations:** 1 Luddy School of Informatics, Computing, and Engineering, Indiana University Bloomington, 107 S Indiana Ave, Bloomington, 47404 USA; 2 Applied Data Science Department, San Jose State University, San Jose, 95192 USA; 3 Computer Engineering Department, Siirt University, 56100 Siirt, Turkey

**Keywords:** scRNA, Imputation, Single-cell, Dropout event, Downstream analysis, Next generation sequencing

## Abstract

**Background:**

In recent years, the introduction of single-cell RNA sequencing (scRNA-seq) has enabled the analysis of a cell’s transcriptome at an unprecedented granularity and processing speed. The experimental outcome of applying this technology is a $M \times N$ matrix containing aggregated mRNA expression counts of *M* genes and *N* cell samples. From this matrix, scientists can study how cell protein synthesis changes in response to various factors, for example, disease versus non-disease states in response to a treatment protocol. This technology’s critical challenge is detecting and accurately recording lowly expressed genes. As a result, low expression levels tend to be missed and recorded as zero - an event known as dropout. This makes the lowly expressed genes indistinguishable from true zero expression and different than the low expression present in cells of the same type. This issue makes any subsequent downstream analysis difficult.

**Results:**

To address this problem, we propose an approach to measure cell similarity using consensus clustering and demonstrate an effective and efficient algorithm that takes advantage of this new similarity measure to impute the most probable dropout events in the scRNA-seq datasets. We demonstrate that our approach exceeds the performance of existing imputation approaches while introducing the least amount of new noise as measured by clustering performance characteristics on datasets with known cell identities.

**Conclusions:**

ccImpute is an effective algorithm to correct for dropout events and thus improve downstream analysis of scRNA-seq data. ccImpute is implemented in R and is available at https://github.com/khazum/ccImpute.

## Introduction

In recent years the development of scRNA-seq technology has enabled a more thorough analysis of how transcriptome expression varies among different cells [1–9] responding to various physiological conditions and external environmental changes [10–13]. Generally, RNA-seq measures the transcription of specific genes by performing reverse-transcription on RNA molecules to transform them into a library of complementary DNA (cDNA) fragments. The cDNA fragments are then sequenced using high-throughput sequencing technology and aligned to a reference genome or transcriptome used to create an expression profile (counting the number of recorded reads) of the genes [14]. Ideally, the number of recorded reads should correspond to the number of mRNA molecules present in the cell; however, the amount of cDNA amplification, the sequencing depth, and the efficiency of the capture and reverse-transcription steps greatly influence that correspondence. Differences in these technical factors across the sequenced cells can and typically do, therefore, partially obscure the relationship between the number of reads and the expressed gene’s actual molecular counts.

As a result, the major challenge with a dataset produced by scRNA-seq is how to deal with a high degree of noise [15–18]. Some noise is due to the inefficiency of mRNA capture and its consequence of using amplification techniques relying on only a small amount of available RNA in a single cell. Furthermore, amplification techniques are prone to distortion and contamination [19–21]. More specifically, reverse transcription and cDNA amplification steps miss some mRNAs and, as a result, there are missing transcripts in the subsequent sequencing step. Current scRNA-Seq technologies capture a mere 10-40% of transcripts in a given cell [22]. The scRNA-seq capture missing an expressed gene is known as a ’dropout’ event. Some literature distinguishes true zero expression events from dropouts by assuming that non-expressed genes would be commonly observed in other cells from a given population with moderate or high expression [23], but this often is an oversimplification, and tools built upon it may introduce bias. This bias and others, which include modifying non-zero values, have introduced a general reluctance to use imputation tools to analyze the scRNA-seq data [24]. However, dropout events cause lower-than-expected performance when employing traditional data mining algorithms since most of these algorithms do not work well with missing values and sparse (zero-inflated) data. Imputing these dropout values without introducing new bias becomes essential in scRNA-seq data analysis.

Many current approaches have been proposed to address the issue of dropout events. Zero-Inflated Factor Analysis (ZIFA) adopts a factor analysis (FA) approach for dimensionality reduction to work with a zero-inflated model. Like FA, ZIFA’s data generation process assumes that cell sub-types initially exist as points in a latent (unobserved) low-dimensional space. Next, these points are projected as points in a latent high-dimensional space corresponding to the gene expression matrix using linear transformation and added Gaussian-distributed noise; this allows each measurement to have some probability of being zero using the dropout model that controls the influence of the latent distribution expression values. [25]. Markov Affinity-based Graph Imputation of Cells (MAGIC) [22] assumes that the denoised data’s underlying structure is a manifold (meaning only locally the distance is Euclidean) and imputes the missing expression values by sharing information across similar cells based on heat diffusion. This method’s essential step is to create a Markov transition matrix by normalizing single cells’ similarity matrix from a large sample size (often thousands of cells). In the imputation of a single cell, the other cells’ weights come from the transition matrix. The developers suggest that MAGIC can recover gene-gene interactions well on several datasets [22]; however, this technique modifies values of genes unaffected by dropout events which introduces bias and sometimes fails to preserve the actual zero values [26]. DrImpute [27] identifies similar cells using clustering. Imputation is performed by averaging the expression values from similar cells. This process repeats multiple times, followed by averaging those multiple estimations to generate the final imputation. scImpute [26] evaluates which values are caused by dropout events in data and performs imputation only on values that are likely to be dropouts. First, scImpute learns each gene’s dropout probability in each cell based on a mixture model. Next, it imputes the most probable dropout values in a cell by gathering information about the same gene from other similar cells only using genes predicted to be unaffected by the dropout events. mcImpute [28] is an imputation algorithm for scRNA-seq data that models gene expression as a low-rank matrix and estimates the dropout values in the process of recovering the full gene expression data from sparse single-cell data by iteratively applying soft-thresholding on the singular values of scRNA-seq data. The most distinguishing feature of mcImpute is its lack of assumptions about gene expression distribution. Deep count autoencoder(DCA) [29] builds an auto-encoder to model the distribution of the genes using a zero-inflated negative binomial prior. An autoencoder consists of two main parts: an encoder that maps the expression data into the compressed model and a decoder that maps the compressed model to a reconstruction of the expression data. The compression is intended to filter out the noise present in the data, and performs well in practice as long as enough data is available. DeepImpute [30] is a neural network (NN) based approach that constructs multiple NN models to impute the dropout events using divide and conquer through dropout layers and loss functions to learn patterns in the data.

This work takes advantage of the effectiveness of consensus clustering as a similarity measure and introduces the ccImpute algorithm that utilizes this similarity measure to impute the dropout events in the scRNA-seq data effectively. We compute the consensus matrix using an approach inspired by the SC3 algorithm [31]. However, rather than using three different distance measures, two dimensionality reduction techniques: Principal Component Analysis (PCA) [32] and Laplacian eigenvalues [33] found in the original algorithm, we utilize weighted Spearman distance measure followed up by PCA dimensionality reduction with data subsets chosen based on relative principal component variance for larger datasets. Our approach results in substantial speedup and improvement of the similarity measure. Next, we let the most similar cells as established by a consensus matrix cast weighted votes to determine whether a given value is a dropout or a true zero. This is followed by computing the dropout events’ values as the weighted mean of most similar cells using a linear equation solver. We empirically show that our approach successfully recovers both linear and non-linear patterns in the data while introducing the least bias. We demonstrate that our approach has a polynomial runtime that compares favorably to imputation algorithms with polynomial (DrImpute, DCA, DeepImpute) and exponential runtime (scImpute). The runtime performance of our approach is only second to MAGIC (polynomial runtime). Further, recent results from applying mini-batch K-means on scRNA-seq [34] and the possibility of using a more efficient centroid selection scheme than random restarts [35] can improve the runtime performance making ccImpute even more suitable for big datasets.

## Results

### Overview of the ccImpute algorithm

**Fig. 1:**
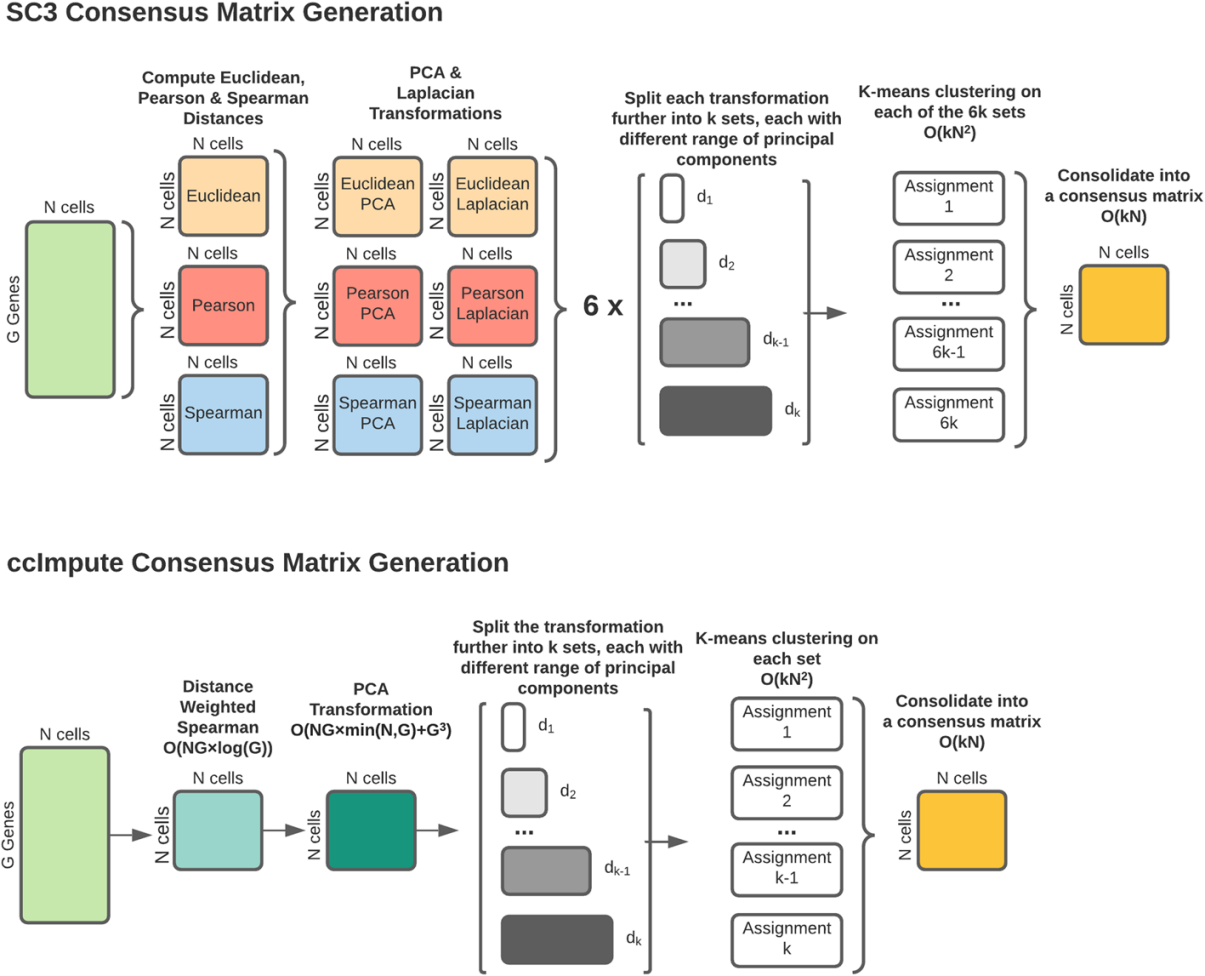
The approach for computing the consensus matrix. The top portion corresponds to the original SC3 algorithm, and the bottom corresponds to the modified version used in our approach. The main difference is using a single more complex distance function along PCA reduction rather than three separate distance functions with two separate dimensionality reduction approaches

ccImpute relies on a consensus matrix to approximate how likely a given pair of cells is to be clustered together and thus considered to be of the same type. Both the general technique and success of SC3 inspired our approach in generating a consensus matrix. SC3 computes Pearson, Spearman, and Euclidean distance measures between cells followed by linear PCA and non-linear Laplacian dimensionality reductions, resulting in six data transformations. Each of these is further split into subsets, each having a different range of reduced dimensions. Each subset is then clustered using the K-means algorithm, and the results of all runs are then aggregated into a consensus matrix. We have experimentally determined (see Additional file 1: supplementary material Section 2, and supplementary Tables 1, 2 and 3) that the weighted Spearman measure alone yields better imputation results than the combination of unweighted Euclidean, Spearman, and Pearson measures. This can be attributed to the fact that introduced weights emphasize the genes that are less affected by the dropout events. Thus the new measure reduces the impact of closeness based on zero values in poorly expressed genes. Finally, we have observed that the use of Laplacian non-linear data dimensionality reduction does not improve performance with the new measure. The graphical representation of the differences in generating a consensus matrix between the two approaches is shown in Fig. 1.

Consequently, we lower the number of data transformations (by a factor of six) as the quality of the consensus matrix is improved. Once the consensus matrix is computed, the values are processed to remove noise and prepared for further matrix operations. Dropout *vs.* true zero values are distinguished by having the cells vote. This is followed by computing the values determined to be dropouts as a weighted mean of its neighbors with weights derived from the consensus matrix. The gene expression counts of neighbors may change as a result of imputation. We leverage an efficient linear equation solver to effectively model the change.

More detailed algorithm elements are outlined in the methods section. In the following subsections, we describe the performance characteristics of ccImpute.

### ccImpute is effective in improving downstream analysis

**Fig. 2:**
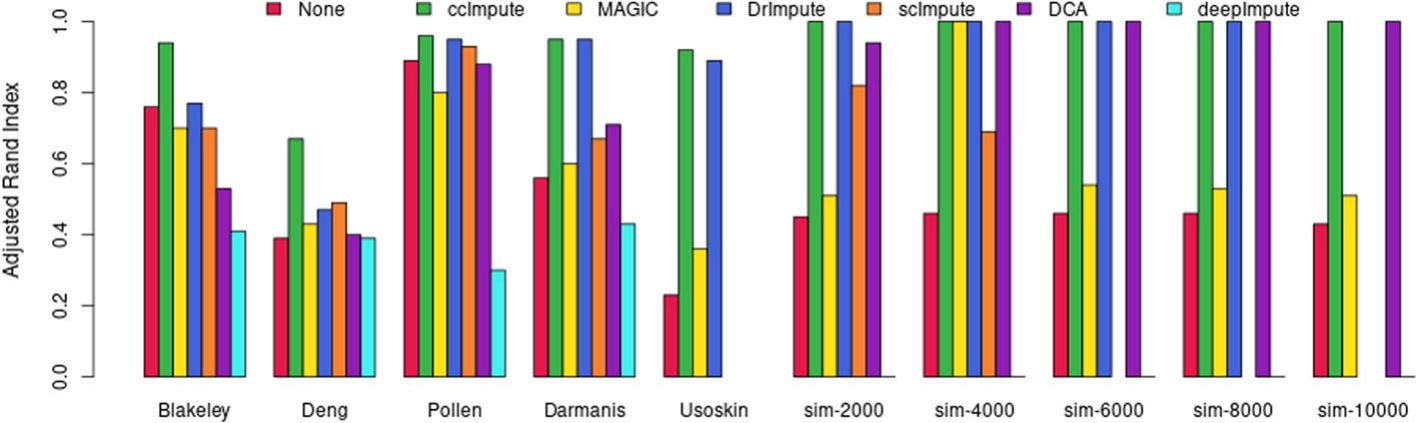
The Adjusted Rand Index (ARI) scores of K-means clustering on PCA reduced scRNA datasets vs. imputation method. Clustering performance is a strong indicator of improved downstream performance, as long as the data is not heavily biased due to imputation. PCA is a linear technique, and this metric aims to measure the impact of the imputation on correcting the linear patterns in the data. The range of possible values is in the interval $[-1,1]$, with a higher value indicating better performance. ccImpute is the best performing approach on all datasets. scImpute, DCA, and DeepImpute only work with raw unnormalized datasets and cannot impute the Usoskin dataset. Further, scImpute and DrImpute timed out on the larger datasets

**Fig. 3:**
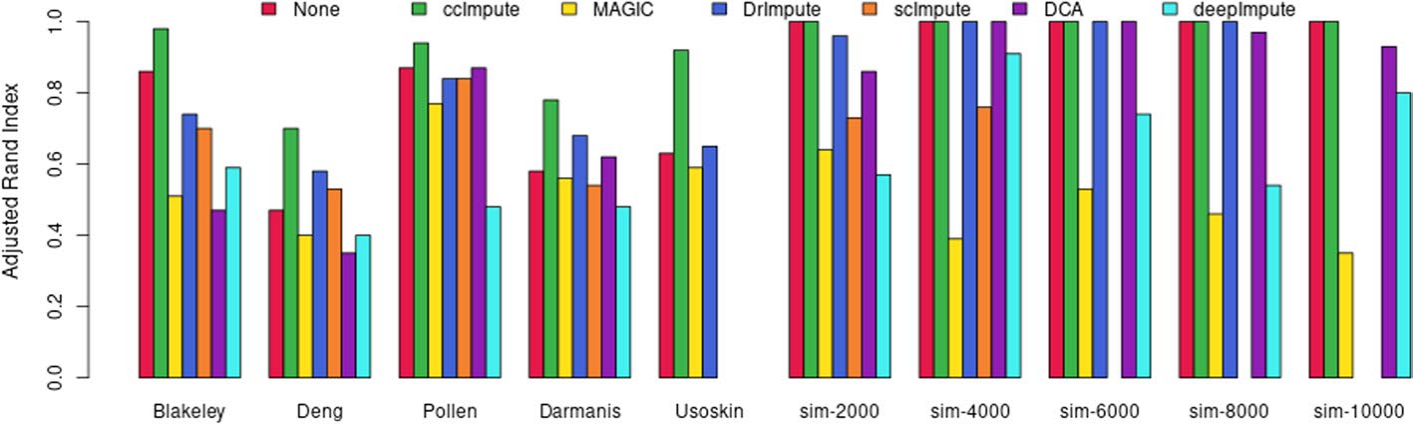
The Adjusted Rand Index (ARI) scores of K-means clustering on t-SNE reduced scRNA datasets vs. imputation method. Clustering performance is a strong indicative of improved downstream performance, as long as the data is not heavily biased as a result of imputation. t-SNE is a non-linear technique, and this metric aims to measure impact of the imputation on correcting the non-linear patterns in the data. The range of possible values is in interval $[-1,1]$, with higher value indicating better performance. ccImpute is the best performing approach on all datasets, and the only algorithm that did not hurt the performance of t-SNE algorithm on any of the datasets. scImpute, DCA, and DeepImpute only work with raw unnormalized datasets and cannot impute the Usoskin dataset. Further, scImpute and DrImpute timed out on the larger datasets

We tested how well ccImpute can improve the quality of downstream analysis by measuring the difference in how well dimensionality reduction techniques, such as PCA(linear) and t-SNE (non-linear)–both known to be significantly affected by the presence of the dropout events–reduce the data while preserving the data characteristics. We compared the quality of K-means clustering on the reduced data using the Adjusted Rand Index (ARI) against the known labels of the datasets. This allows measuring to what degree imputation affects the simplest task of grouping the like cells. Our ten scRNA-seq datasets are comprised of five publicly available datasets and five synthetically generated datasets. We compared ccImpute with five other state-of-the-art algorithms: MAGIC, DrImpute, scImpute, DCA, and DeepImpute. ccImpute’s imputation resulted in the best improvement of K-means clustering quality as measured by ARI scores on data reduced both by PCA and t-SNE as summarized in Figs. 2 and 3.

### ccImpute distorts the data the least

**Fig. 4:**
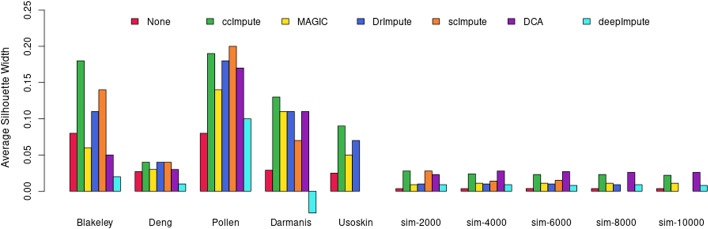
The bar plots show Silhouette widths values across the datasets with clustering assignments corresponding to the labels and Euclidean distances between imputed cells data. This metric shows if the imputation of the zero values exclusively has improved the separation of the cell data in the multidimensional space. The range of possible values is in the interval $[-1,1]$, with a higher value indicating better performance. ccImpute is the best performing approach overall, with scImpute performing slightly better on the Pollen dataset and DCA on some simulated datasets showing varied values where constant values are expected due to the characteristics of the simulated data. scImpute, DCA, and DeepImpute only work with raw unnormalized datasets and cannot impute the Usoskin dataset. Further, scImpute and DrImpute timed out on the larger datasets

**Fig. 5:**
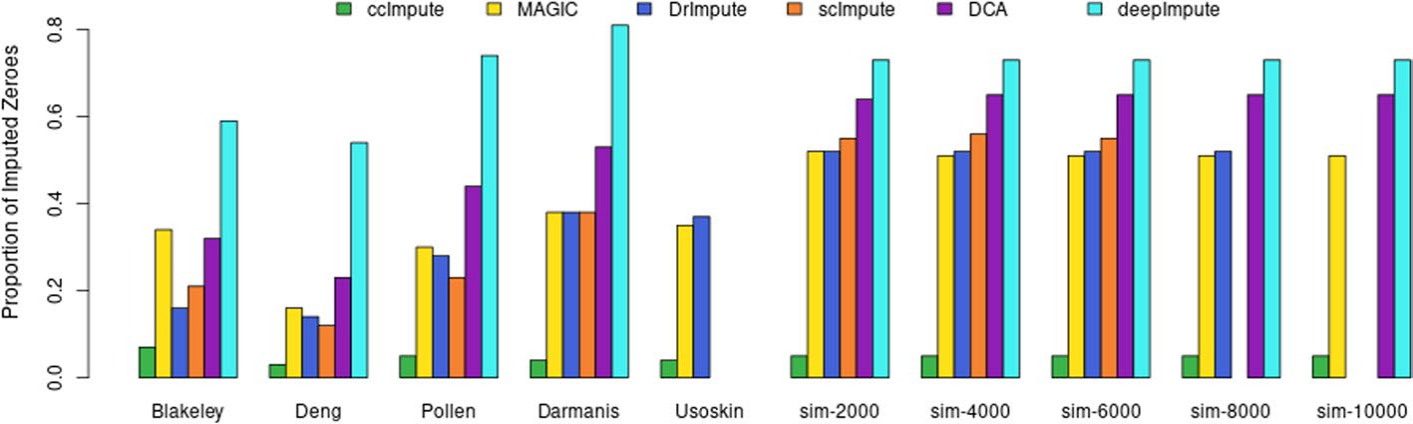
Zero values make up a large portion of the scRNA-seq expression counts. It’s expected that a significant portion of these values corresponds to true zero expression or is zero since there is not enough information to impute any other value credibly. The bar plots show the proportion of zero count values that are replaced by a value of 0.5 or higher due to imputation. Many imputation approaches modify a significant fraction of zero values without being correlated with improved downstream analysis performance. In other words, these approaches introduce more bias, which negatively impacts the scRNA-seq expression data analysis. ccImpute outperforms the competitors in downstream analysis while modifying much fewer values. scImpute, DCA, and DeepImpute only work with raw unnormalized datasets and cannot impute the Usoskin dataset. Further, scImpute and DrImpute timed out on the larger datasets

**Fig. 6:**
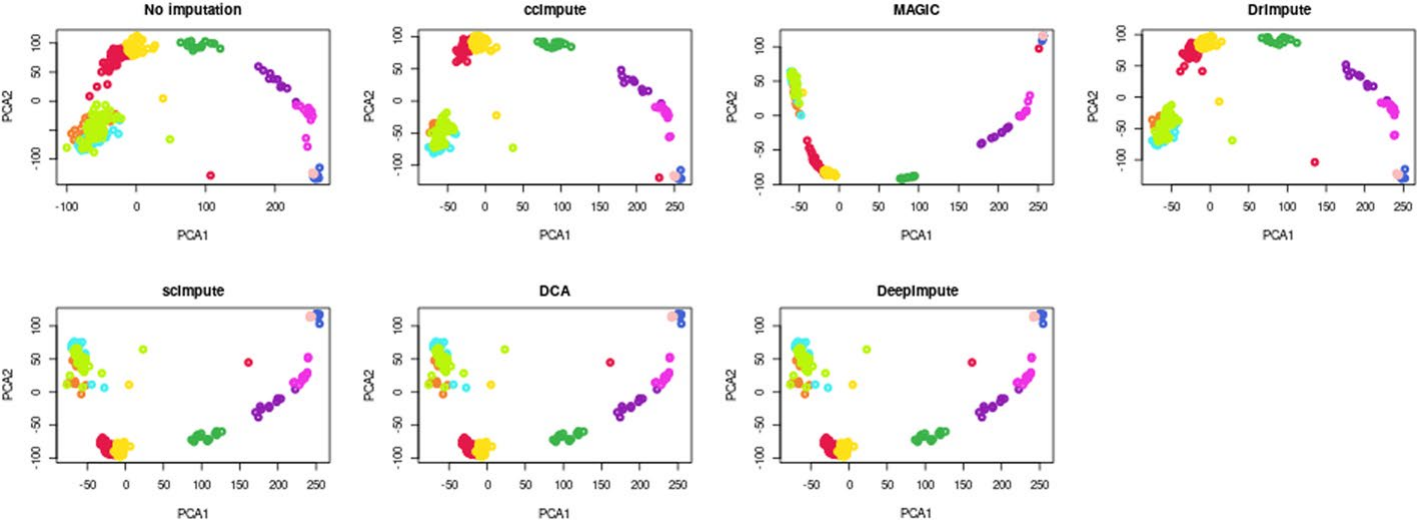
PCA graph PC2 vs. PC1 for the Deng dataset for all the compared imputation algorithms. Among all the considered imputation approaches, ccImpute shows the most resemblance to the non-imputed data while having the best separation between different groups of cells and modifying the least amount of values

Whether actual or dropout values in scRNA-seq datasets, most of the matrix is populated by zeroes. Thus, intuitively only a fraction of zeroes can be reliably recognized as dropout values and imputed since the estimation of dropout values has to happen given the limited remaining data. Given the hesitancy to use imputation tools in the research community due to introduced bias, we evaluate the impact of imputation algorithms on changing expression values. As shown in Fig. 4, ccImpute separates the clusters most successfully when taking into consideration imputation of zero values exclusively and comparing average silhouette widths of clustering assignments based on actual labels. As shown in Figs. 2, 5 and 6, ccImpute superior improvement in clustering quality and cluster separation is achieved while modifying the least amount of values. This demonstrates that the dropout values alone are the principal cause for the degradation of the downstream analysis, and competing imputation approaches modify more values with inferior outcomes, which suggests artificial bias added to the data.

### ccImpute is a scalable machine learning method

**Fig. 7:**
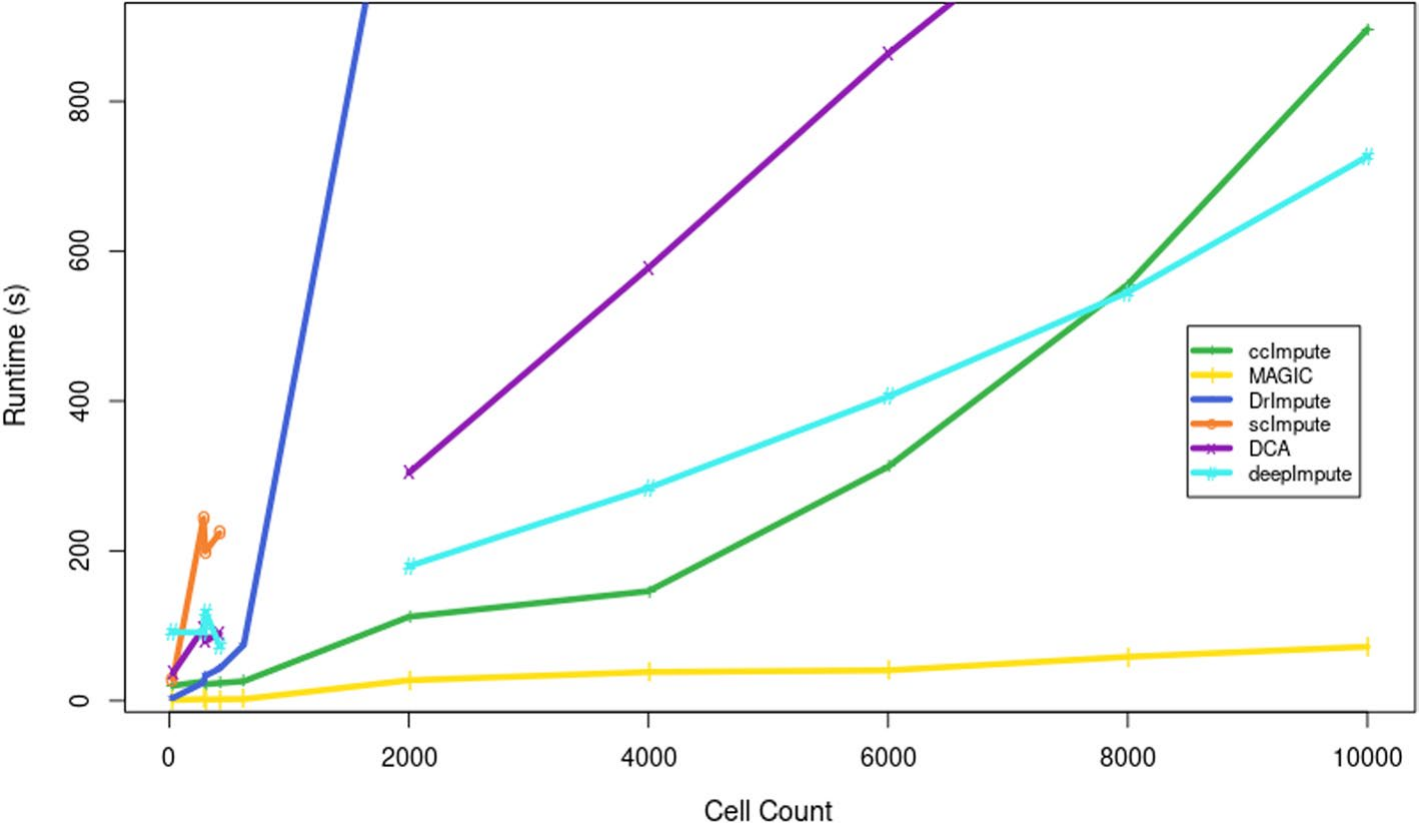
The plot shows runtimes (s) for the imputation algorithms under consideration. DrImpute and scImpute did not scale favorably with data size and timed out at the maximum allotted time of 160 minutes on the simulated datasets. MAGIC was the fastest method with polynomial runtime complexity. ccImpute was the second fastest on datasets with less than 8000 cells, with DeepImpute taking the second fastest after 8000 cells. This shows ccImpute has competitive runtime, and with further optimizations such as swapping K-means for a faster clustering algorithm, this approach may take the lead in both runtime performance and quality of imputation

ccImpute has $O(N^3)$ runtime where *N* corresponds to a cell sample size. Since most of the operations in our approach are linear algebra operations involving matrices, and such operations are already optimized in most computing environments due to the presence of BLAS and LAPACK libraries, ccImpute runs much faster in practice. As seen in Fig. 7 the runtime of ccImpute compares favorably to imputation algorithms with polynomial (DrImpute, DCA, DeepImpute) and exponential runtime (scImpute). The runtime performance of our approach is only second to MAGIC (polynomial runtime). Furthermore, a major bottleneck of our approach is the K-means algorithm runs needed for computing the consensus matrix. However, our approach is not tied to any specific clustering algorithm, and further optimizations are possible to lower the runtime. One of such modifications mentioned earlier is mini-batch K-means with a more intelligent cluster selection scheme. Concerning hardware, approaches that rely on TensorFlow (DCA, DeepImpute) may take advantage of GPU acceleration and, with proper setup, will considerably exceed the speeds of the parallel CPU setup we used in our experiments. Our approach is an excellent candidate for GPU acceleration and is expected to have a competitive run-time with GPU acceleration implementation.

## Discussion

Dropout events present a challenge in downstream analysis of the scRNA-seq data. This work presents an imputation method based on the existing success of consensus clustering. We demonstrate that our method deals with the problems of dropout events in scRNA-seq better than any of the state-of-the-art approaches by improving K-means clustering accuracy and like cell separation after linear (PCA) and non-linear dimensionality reductions (t-SNE), both affected by the presence of dropout events. Our technique allows a formal assessment of the degree noise affects clustering assignments. Thus, we can focus on relationships between cells that are not affected much with the presence of a bit of noise. We use this information in the form of a consensus matrix to establish what values are to be imputed by employing weighted voting. This is followed by a weighted mean imputation of the values determined to be dropout events. We demonstrate experimentally that our method not only improves the performance of downstream analysis best, but also does with least bias introduced to the count values. We also include an additional set of results in the Additional file 1: supplementary material Section 1 and supplementary Figures 1 and 2 that confirm our findings in light of supervised machine learning.

## Methods

### Data pre-processing

Five experimentally obtained (Blakeley, Pollen, Deng, Darmanis, Usoskin) and five synthetically generated scRNA-seq datasets with details summarized in Table 1 were used to benchmark the performance of our imputation approach. Pollen and Blakeley have the highest quality of labeling since their labels depend on either experimental conditions or cell lines. Labels for Usoskin and Darmanis were derived computationally with expert feedback. Finally, Deng consisted of the same cell type in different developmental stages. For the Darmanis dataset we removed the cells that were labeled as hybrids. Any of the genes that were not expressed in any cells were removed. The normalized matrix *N* was computed by dividing raw read counts for each cell by a cell’s total read count, then multiplied by a scale factor of 1, 000, 000, and finally, the log-transformed matrix *T* was computed using $(log_2(X+1)$). The synthetically generated data had four cell populations with uneven distributions (.3, .2, .35, .15) and only dropout noise added to all cells without any additional noise.

**Table 1:** The scRNA-seq datasets used to benchmark the quality of imputation. k represents the number of cell clusters reported in the original study

Dataset	# Samples	# Features	k	Cell Origin	References
Blakeley	30	16862	3	Human blastocyst samples	[43]
Deng	286	18884	10	Stages of mouse pre-implementation development	[44]
Pollen	301	23730	11	Human cell lines	[5]
Darmanis	420	21516	8	Human cortical samples	[45]
Usoskin	622	19532	4	Mouse lumbar	[1]
sim-n	n	20000	4	Splatter synthetically generated data	[46]

The datasets generated and/or analysed during the current study are available in the Github repository, https://github.com/khazum/ccImputeDatasets

### Weighted Spearman distance

As the first step, each dataset was reduced to a $N \times N$ weighted Spearman distance matrix with weights corresponding to genes’ variances. This reduction began by ranking the gene expression vectors, then computing the Pearson correlation coefficient on those vectors. The rank of the $j^{th}$ element is:

$$\begin{aligned} rank_j = a_j + b_j \end{aligned}$$

where $a_j$ is the sum of all weights *W* less than or equal to the ranked outcome $\beta _j$:

$$\begin{aligned} a_j = \sum _{i=1}^{n}w_i \, f(\beta _i < \beta _j) \end{aligned}$$

where *f*() is a function that returns 1 if $\beta _i < \beta _j$ is true, and 0 otherwise. The term $b_j$ deals with ties. The vector of tied ranks is $v = (a_j + w^{'}_1, a_j + w^{'}_{1} +w^{'}_{2}, \ldots , a_j + \sum _{k=1}^{n} w^{'}_k)^{T}$ with $W^{'}$ being a vector containing the weight of tied units. The mean value of the tied values vector will depend on the ordering of the weights. To solve this problem, the overall mean of all permutations of the weights is calculated using:

$$\begin{aligned} b_j = \frac{n+1}{2}{\bar{w}}_{j} \end{aligned}$$

where ${\overline{w}}_{j}$ is the mean weight of all tied units. After ranking vectors *X* and *Y*, they are plugged into weighted Pearson correlation coefficient formula:

$$\begin{aligned} \rho _{Pearson} = \frac{\sum _{i=1}^{n}[w_i(x_i-{\bar{x}})(y_i-{\bar{y}})]}{\sqrt{\sum _{i=1}^{n}(w_i(x_i-{\bar{x}})^2)\sum _{i=1}^{n}(w_i(y_i-{\bar{y}})^2}} \end{aligned}$$

### Principal component analysis (PCA)

Principal Component Analysis (PCA) transformation was computed on weighted Sperman Distance matrix with the variables shifted to be zero centered and scaled to have unit variance. PCA [36] is a statistical data (dimensionality) reduction procedure that takes ${\mathcal {D}} =\{x_1,\ldots ,x_n\}\in \Re ^m$ as an input and returns $\hat{{\mathcal {D}}}$, which is a *d*-dimensional representation of ${\mathcal {D}}$ where $d < m$. $\hat{{\mathcal {D}}}$ is found as follows:

1. Center ${\mathcal {D}}$ (Mean Normalization):
  1 $$\begin{aligned} \hat{{\mathcal {D}}} = \left( I -\frac{e e^t}{n}\right) \, {\mathcal {D}} \end{aligned}$$
  where *I* denotes n$\times$n identity matrix and $e = (1,\ldots ,1)\in \Re ^n$.
2. Compute Singular Value Decomposition of $\hat{{\mathcal {D}}}$:
  2 $$\begin{aligned} \hat{{\mathcal {D}}} = \sum \limits _{i=1}^r \sigma _i u_i v_i^t \end{aligned}$$
  where $\sigma _1 \ge \cdots \ge \sigma _r > 0$ are strictly positive singular values of $\hat{{\mathcal {D}}}$ and $u_1,\ldots ,u_r$ and $v_1,\ldots ,v_r$ are corresponding left and right singular vectors respectively. *r* is the rank of matrix ${\mathcal {D}}$.
3. Construct *d*-dimensional ($d<r$) principal component representation of ${\mathcal {D}}$ (Final $\hat{{\mathcal {D}}}$):
  3 $$\begin{aligned} \hat{{\mathcal {D}}} = \Big [ \sigma _1 u_1 |\cdots | \sigma _d u_d \Big ] = \begin{bmatrix} \hat{\delta _1^t} \\ \vdots \\ \hat{\delta _n^t} \end{bmatrix} \end{aligned}$$

In PCA the data ${\mathcal {D}}$ is transformed to a lower dimensional coordinate system based in the co-variance matrix of ${\mathcal {D}}$. This transformation can be done (i) Linearly or (ii) Non-linearly. In this work, we refer to linear PCA as PCA. In PCA a set of linearly correlated variables is mapped into a new set of linearly uncorrelated variables using an orthogonal linear mapping. These new variables, called principal components, are a linear combination of the original variables. Principal components are orthogonal and decrease in the amount variance in the originals they account for. The first component captures most of variance, the second less, and so on until all the variance is accounted for. Kernel PCA (nonlinear PCA is) non-linearly maps the data using the kernel trick [37, 38].

Geometrically, given *m*-dimensional data, PCA fits a *d*-dimensional ellipsoid to the data where principal components are axes of the ellipsoid. Small and long axes represent small and large variances, respectively. The data is reduced by omitting the small axes. PCA performs well when when the data is high dimensional and the variables are correlated. Correlation is an indication of redundancy between the variables. Additionally, PCA is sensitive to the relative scaling of the original variables.

### *K*-means clustering algorithm

Clustering [39, 40] a descriptive data analytics method and aims to partition data into blocks such that the difference between data in a block is as small as possible. *K*-means (KM) is the most commonly used clustering algorithm despite its simplicity and almost 60-year lifetime [41, 42]. KM clusters in a greedy fashion. Assume each data point ${\mathbf {x}}$ is a vector over $\Re ^m$. Then KM partitions ${\mathcal {D}}$ ($\mathcal {|D|} =n)$ into a set of non-empty blocks $X_0, X_1, \ldots X_k$ such that $\cup _i X_i = {\mathcal {D}}$ and $X\cap Y = \emptyset$ for all disjoint sets $X,Y\in {\mathcal {D}}$. A distance metric $d:{\mathcal {D}}^2 \rightarrow \Re _{\ge 0}$ measures closeness of data, and the most commonly used is Euclidean distance ($L_2$ norm).

The KM algorithm takes as input data ${\mathcal {D}}$, the number of clusters *k*, and a distance metric *d* and produces a set of centroids $C_1, C_2, \ldots , C_\ell$ that effectively partition ${\mathcal {D}}$. After randomly initializing *k* centroids $\{C_1, C_2, \ldots C_k\}$, KM iteratively runs as follows:

1. Assign each data datum to nearest centroid:
  $$\begin{aligned} {\text {argmin}}_{j=1,\ldots k} ||C_j - {\mathbf {x}}||,\ i = 1,\ldots n \end{aligned}$$
2. Update centroids $$\{C_1, C_2, \ldots C_k\}$$:
  $$\begin{aligned} C_{j} = \left( \frac{1}{|C_j|}\right) \sum _{\mathbf {x_i} \in C_j }^{|C_j|} \mathbf {x_i} \end{aligned}$$

The simplest approach to updating centroids is calculating the average of the block. Iteration continues until the set of centroids is stable; in other words, convergence is guaranteed in a finite number of steps by showing that for some non-negative error function

$$\begin{aligned} \sum _{i=1}^{n}\sum _{j=1}^{k}{||\mathbf {x_i} - C_j||^2_2 } \end{aligned}$$

is monotonically decreasing during each iteration.

The initialization of the starting set of centroids greatly affects KM’s performance. While in theory convergence is assured, achieving this in practice is another matter. Often, the iterate is part of the stopping condition. While relatively easy to understand and implement, the strategy has a significant weakness in that by simply increasing *k*, error will be made smaller without any apparent value to the output of the algorithm itself. Given that the algorithm is so simple, finding other limitations in KM is not difficult. For example, while many metrics are available Euclidean distance has historically been the first choice. Recent work showing a more general class of distance, Bregman divergences, has proven useful in signal processing and speech recognition. This allows, under some conditions, a broadening of the data amenable to KM. However, as a corollary to its sensitivity to outliers, when ${\mathcal {D}}$ becomes dense, convergence severely slows. While this means KM is in practice now *O*(*nmki*), it also requires more discriminatory power–even ignoring the curse of dimensionality, a general problem all algorithms face.

### Computing consensus matrix

We used PCA to transform the weighted Spearman distance matrix, that was further split into several sub-datasets each corresponding to a different number of most significant principal components. Our goal was to produce sub-datasets to measure aggregated KM clustering similarity with respect of varying amount of noise added. For datasets with 500 cells or more, we started with all principal components that were equal or less than 0.01 relative variance and kept adding sub-datasets until reaching the number of principal components with 0.008 relative variance or less. For small datasets, this was done using a specific range following the sequence $[0.04N, 0.04N+1,\ldots , 0.07N-1, 0.07N]$, since the relative variance values in this scenario hold little statistical significance. The total number *M* of the subsets was limited to 15 by selecting every other *floor*(*M*/15) sub-dataset. This was followed by KM clustering on each sub-dataset with $1e+09$ max iterations and 1000 random restarts. The number of random restarts was reduced to 50 for datasets with more than 2000 cells. For each individual clustering result a binary similarity matrix was constructed from the corresponding cell labels: if two cells belong to the same cluster, their similarity is 1, otherwise the similarity is 0. A consensus matrix *C* is calculated by averaging all similarity matrices. This process is summarized in Fig. 1.

### Imputing dropout events

The pseudocode of the ccImpute is outlined in Algorithm 1. As the first step in lines 2 and 4 the influence of the cell itself and all entries below a threshold value in the consensus matrix are removed to lower the influence of unlikely cell clustering assignments. This threshold value should be lower for higher quality clustering approaches and higher for lower quality approaches. In line 6: the remaining entries are reweighed so that each row values become a probability vector, and the resulting non-valid numbers are replaced with 0 in line 8. In lines 11-14, each cell casts a weighted vote to decide if a given value is a dropout. A cell’s vote magnitude is its consensus value with the cell it is voting on. If the cell’s gene entry value is non-zero, the vote is positive and negative otherwise. If the sum of votes is above zero, the gene count value of a cell is considered a dropout and needs to be imputed. Otherwise, it is left unchanged. Next, the values determined to be dropouts are computed as a weighted mean of its neighbors with weights derived from the consensus matrix. Since some of the dropout values to be imputed depend on other not yet imputed values, this problem is reformulated as a system of linear equations in lines 18-33. In line 35, the values of dropouts are computed using a linear equation solver. This is followed by replacing the dropout values in the original matrix with newly computed values in lines 36-38.

### Evaluation

To evaluate the performance of the ccImpute algorithm, we compared how well the proposed imputation improves the quality of the PCA/K-means clustering and t-SNE/K-means clustering as compared to other imputation approaches (DrImpute, scImpute, DCA, MAGIC and DeepImpute comparing the Adjusted Random Indices. We further compared the separation of the data points corresponding after each imputation method using average Silhouette widths with clustering assignments corresponding to true labels and distances corresponding to Euclidean distances between the data points. We also measured how many zero entries have changed due to imputation. Since some approaches have added noise to the zero entries, we considered any imputed non-log transformed values of less than 0.5 to be zero. This was recorded as a percentage of total zero values. Finally, we evaluated the runtime versus size of the dataset for each of the algorithms. Each experiment was repeated 100 times for datasets with less than 2000 cells and 10 times otherwise.

### Evaluation metrics

#### Adjusted rand index ARI

Given two clustering assignments *X* and *Y* of *n* data points, the Rand Index (RI) is:

$$\begin{aligned} RI = \frac{a+b}{a+b+c+d} \end{aligned}$$

where *a* corresponds to the pair of data points that are in the same cluster in *X* and *Y*, *b* corresponds to pair of data points that are in different clusters in *X* and *Y*, *c* corresponds to pair of data points that are in the same cluster in *X* but in different clusters in *Y*, and d corresponds to pair of data points that are in different clusters in *X* but in the same cluster in *Y*.

Adjusted Rand index (ARI)is adjusted for chance as follows:

$$\begin{aligned} ARI = \frac{(RI - Expected(RI))}{max(RI)-Expected(RI)} \end{aligned}$$

#### Average Silhouette width

For each data point *i* in Cluster $C_I$, the Silhouette Score:

$$\begin{aligned} s(i) = \frac{b(i) - a(i)}{max(a(i),b(i))} \end{aligned}$$

where

$$\begin{aligned} a(i) = \left( \frac{1}{|C_i| - 1}\right) \sum _{j \in C_I, i \ne j}d(i,j) \end{aligned}$$

with *d*(*i*, *j*) being a a distance metric between *i* and *j*, and for $|C_1|>1$:

$$\begin{aligned} b(i) = \frac{b(i)-a(i)}{max(a(i),b(i))} \end{aligned}$$

and for $|C_1|=1$, $s(i)=0$. The average Silhouette width ${\bar{s}}$ over n data points:

$$\begin{aligned} {\bar{s}} = \frac{\sum ^n_{i}s(i)}{n} \end{aligned}$$

### Experimental platform

All experiments were run on a general-purpose Lenovo NeXtScale nx360 M5 compute node equipped with two 12-core Intel Xeon E5-2680 v3 CPUs and four 480 GB solid-state drives. The number of cores available for computation was set to 16. The R version 4.1.1 was compiled with the Intel compiler version 2021.4.0 using the Intel MKL library to maximize the FLOP performance of matrix operations in our experiments.

### Packages, and input parameters

The SC3 v3.14 package was downloaded from R Bioconductor, and served as foundation to generate the consensus matrix in our approach.

The Rtsne v0.15 R package was used with perplexity=30 for most datasets with exception of Blakeley where perplexity=9 was set to account for low number of samples. This was followed by kmeans core function from stats package v3.6.2 in R with algorithm=Hartigan-Wong and options: iter.max=$10^9$ and nstart=1000 for datasets with less than 2000 cells, and nstart=50 otherwise.

R MAGIC v3.0.0 was used with default settings, and no gene filtering.

DrImpute was installed from the Github repository: gongx030/DrImpute. The data was run without gene filtering step.

scImpute v0.0.8 was installed from Github repository: Vivianstats/scImpute. The algorithm was run with default settings, and number of cores set to 15.

DCA v0.3.1 was installed via preferred installer program in python. The data was imputed with defaults settings.

DeepImpute Release 1 was installed via preferred installer program in python. The algorithm was run with default settings.

Splatter v1.18.2 was installed from Bioconductor. The data was generated with groups setting, group membership probabilities (.15, .20, .30, .35),built-in dropout setting applied to all cells, and no batch noise added.
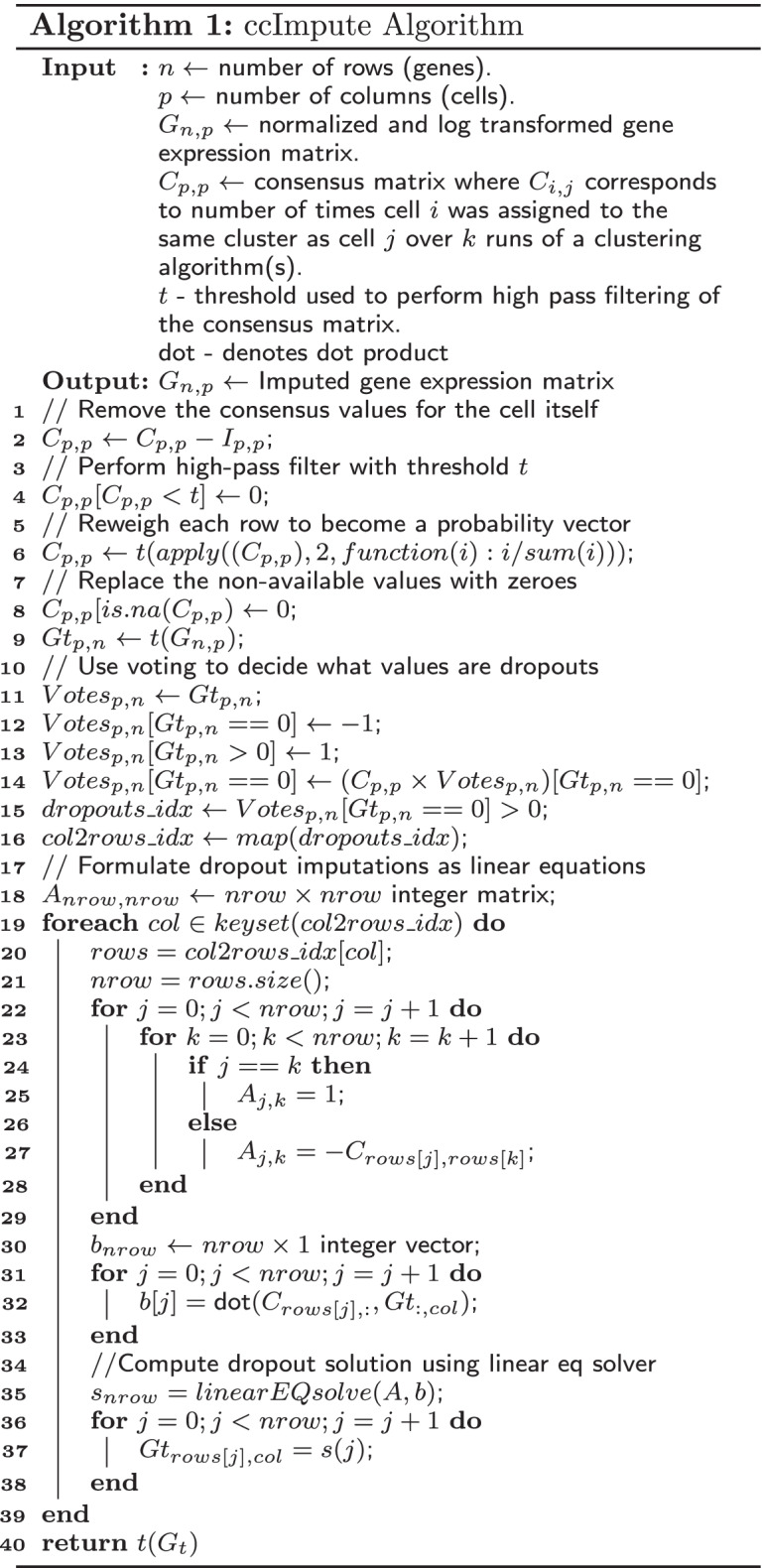


## Supplementary Information

**Additional file 1**. Supplementary Information. A pdf file that contains experimental details and results along with relevant figures and tables omitted from the main paper.

## Data Availability

ccImpute was implemented as an R package, and it is available in the GitHub repository, https://github.com/khazum/ccImpute. The datasets generated and/or analysed during the current study are available in the Github repository, https://github.com/khazum/ccImputeDatasets.
