# Supplementary material for: ccImpute: an accurate and scalable consensus clustering based algorithm to impute dropout events in the single-cell RNA-seq data
Source: BMC Bioinformatics. 2022 Jul 22;23:291. doi: 10.1186/s12859-022-04814-8 (PMC9306045; doi:10.1186/s12859-022-04814-8)
Supplement: Supplementary file 1 — Additional file 1. Supplementary Information. A pdf file that contains experimental details and results along with relevant figures and tables omitted from the main paper. [file 12859_2022_4814_MOESM1_ESM.pdf]

ccImpute: an accurate and scalable consensus clustering based algorithm to impute dropout events in the single-cell RNA-seq data

Supplementary Material

Marcin Malec, Hasan Kurban, Mehmet Dalkilic

1 Evaluating ccImpute imputation in supervised setting

PCA and t-SNE are standard approaches in data reduction and data visualization. Both exhibit impaired performance due to the presence of dropout events in the scRNA-seq data. Here we evaluate improvement in the dimensionality reduction of the scRNA-seq by PCA and t-SNE to 2D space with respect to the imputation approach. To evaluate this, we train a linear SVM on 90% of imputed data and test on the remainder of 10% data. This is repeated ten times with different choices of training and testing data sets in a process known as k-fold cross-validation where $k = 10$. We record the average classification performance on all the test data sets. This is accomplished using the same environment and experimental approach as other experiments presented in the main article. The R packages e1071 (ver. 1.7-9) and caret (ver. 6.0-92) provide linear SVM and k-fold cross validation implementations respectively. The results are summarized in Supplementary Figures 1 and 2. ccImpute shows the best performance for both t-SNE and PCA 2D reductions across all the considered data sets.

Supplemental Figure 1: The bar plots of average accuracy of separating scRNA cell subpopulations in 2D space corresponding to the first two principal components of PCA reduced data vs. imputation method. Classification performance in this scenario is a strong indicator of improved downstream performance - dimensionality reduction and visualization. PCA is a linear technique, and this metric aims to measure the impact of the imputation on correcting the linear patterns in the data. The range of possible values is in the interval $[0, 1]$, with a higher value indicating better performance. ccImpute is the best performing approach on all datasets. scImpute, DCA, and DeepImpute only work with raw unnormalized datasets and cannot impute the Usoskin dataset. Further, scImpute and DrImpute timed out on the larger datasets.

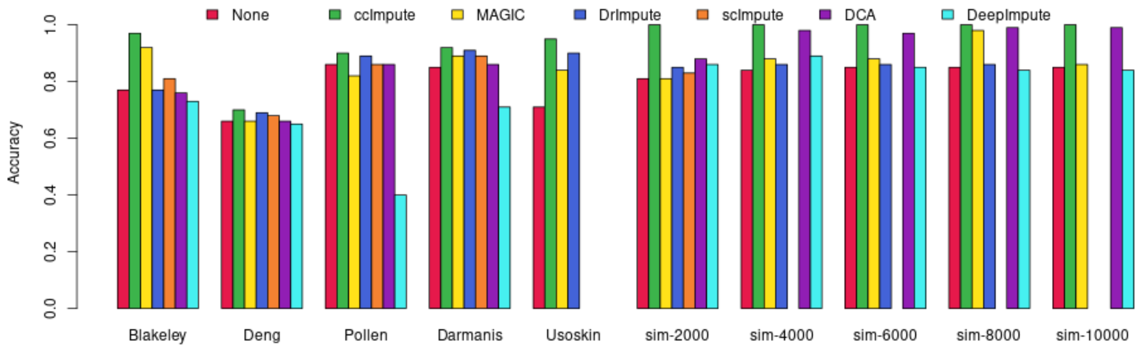

2 Comparison of distance measures performance in ccImpute

The choice of distance measure affects the performance of the ccImpute imputation approach. In all the experiments in the main article, we have used the weighted Spearman distance measure to reduce the original dataset. We have repeated all the experiments on the in vitro datasets and include the results here to demonstrate differences in the performance. The results for unweighted combination of Euclidean, Pearson, and Spearman distance measures are in Supplementary Table 1, unweighted Spearman are in Supplementary Table 2, and weighted Spearman values are in Supplementary Table 3. Weighted Spearman distance measure is the best choice among the measures we have considered.

Supplemental Figure 2: The bar plots of average accuracy of separating scRNA cell subpopulations in 2D space corresponding to the t-SNE reduced data vs. imputation method. Classification performance in this scenario is a strong indicator of improved downstream performance - dimensionality reduction and visualization. t-SNE is a non-linear technique, and this metric aims to measure the impact of the imputation on correcting both the linear and non-linear patterns in the data. The range of possible values is in the interval $[0, 1]$, with a higher value indicating better performance. ccImpute is the best performing approach on all datasets. scImpute, DCA, and DeepImpute only work with raw unnormalized datasets and cannot impute the Usoskin dataset. Further, scImpute and DrImpute timed out on the larger datasets.

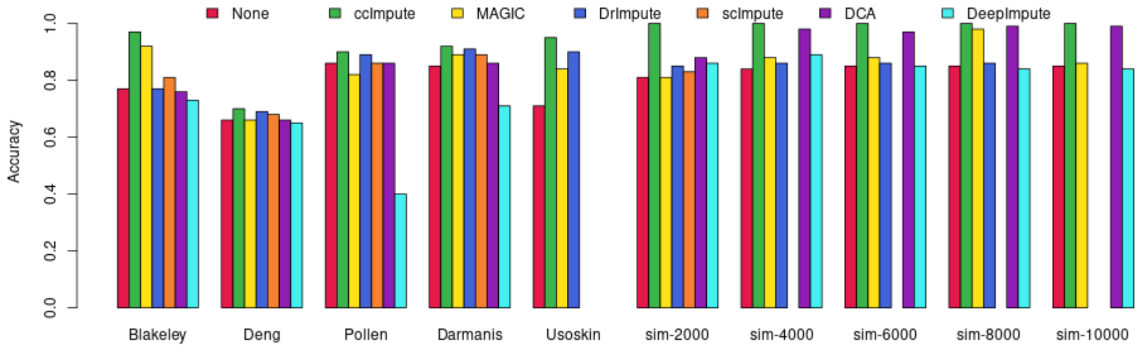

Supplemental Table 1: Choice of distance measure affects the performance of ccImpute imputation approach. This Supplementary Table presents the unweighted combination of Euclidean, Pearson, and Spearman distance measures used to obtain the consensus matrix vs. all the results that correspond to measuring improvement in downstream analysis of scRNA-seq data. The results indicate that the unweighted combination of Euclidean, Pearson, and Spearman distance measures is the third-best choice among the distance measures we have considered.

	**PCA ARI**	**t-SNE ARI**	**Silh. score**	**2-PCA SVM**	**4-PCA SVM**	**t-SNE SVM**	**Mean**
**Blakeley**	0.76	0.70	0.15	0.81	1.00	0.76	0.70
**Deng**	0.49	0.58	0.04	0.70	0.71	0.75	0.55
**Pollen**	0.96	0.88	0.19	0.90	0.96	0.92	0.80
**Darmanis**	0.96	0.74	0.12	0.92	0.96	0.97	0.78
**Usoskin**	0.89	0.90	0.09	0.92	0.95	0.94	0.78
**Mean**	0.81	0.76	0.12	0.85	0.92	0.87	**0.72**

Supplemental Table 2: Choice of distance measure affects the performance of ccImpute imputation approach. This Supplementary Table presents the unweighted Spearman distance measure used to obtain the consensus matrix vs. all the results that correspond to measuring improvement in downstream analysis of scRNA-seq data. The results indicate that the unweighted Spearman distance measure is the second-best choice among the distance measures we have considered.

	**PCA ARI**	**t-SNE ARI**	**Silh. score**	**2-PCA SVM**	**4-PCA SVM**	**t-SNE SVM**	**Mean**
**Blakeley**	0.77	0.90	0.16	0.95	1.00	0.94	0.79
**Deng**	0.50	0.55	0.03	0.69	0.67	0.70	0.52
**Pollen**	0.96	0.90	0.19	0.90	0.96	0.94	0.81
**Darmanis**	0.96	0.80	0.13	0.92	0.97	0.97	0.79
**Usoskin**	0.84	0.85	0.07	0.94	0.94	0.91	0.76
**Mean**	0.80	0.80	0.11	0.88	0.91	0.89	**0.73**

Supplemental Table 3: Choice of distance measure affects the performance of ccImpute imputation approach. This Supplementary Table presents the weighted Spearman distance measure used to obtain the consensus matrix vs. all the results that correspond to measuring improvement in downstream analysis of scRNA-seq data. The results indicate that the weighted Spearman distance measure is the best choice among the distance measures we have considered.

	**PCA ARI**	**t-SNE ARI**	**Silh. score**	**2-PCA SVM**	**4-PCA SVM**	**t-SNE SVM**	**Mean**
**Blakeley**	0.94	0.99	0.18	0.97	1.00	0.99	0.85
**Deng**	0.66	0.71	0.04	0.70	0.82	0.84	0.63
**Pollen**	0.96	0.96	0.18	0.90	0.96	0.93	0.82
**Darmanis**	0.95	0.78	0.13	0.92	0.97	0.97	0.79
**Usoskin**	0.90	0.91	0.08	0.95	0.96	0.94	0.79
**Mean**	0.88	0.87	0.12	0.89	0.94	0.93	**0.77**
